# Male obesity impacts DNA methylation reprogramming in sperm

**DOI:** 10.1186/s13148-020-00997-0

**Published:** 2021-01-25

**Authors:** Sanaz Keyhan, Emily Burke, Rose Schrott, Zhiqing Huang, Carole Grenier, Thomas Price, Doug Raburn, David L. Corcoran, Adelheid Soubry, Catherine Hoyo, Susan K. Murphy

**Affiliations:** 1 Division of Reproductive Endocrinology and Infertility, Department of Obstetrics and Gynecology, Duke University Medical Center, Durham, NC 27713 USA; 2 Department of Biostatistics, Duke University, Durham, 27710 USA; 3 Division of Reproductive Sciences, Department of Obstetrics and Gynecology, Duke University Medical Center, 501 W. Main Street, Suite 510, The Chestefield Building, PO Box 90534, Durham, NC 27701 USA; 4 Duke University Integrated Toxicology and Environmental Health Program, The Nicholas School of the Environment, Duke University, Durham, NC 27708 USA; 5 Center for Genomics and Computational Biology, Duke University Medical Center, Durham, NC 27710 USA; 6 Epidemiology Research Group, Department of Public Health and Primary Care, Faculty of Medicine, KU Leuven University, 2000 Leuven, Belgium; 7 Department of Biological Sciences, Center for Human Health and the Environment, North Carolina State University, Raleigh, NC 27633 USA

**Keywords:** Epigenetics, Methylation, Reprogramming, Sperm, Obesity, TIEGER study

## Abstract

**Background:**

Male obesity has profound effects on morbidity and mortality, but relatively little is known about the impact of obesity on gametes and the potential for adverse effects of male obesity to be passed to the next generation. DNA methylation contributes to gene regulation and is erased and re-established during gametogenesis. Throughout post-pubertal spermatogenesis, there are continual needs to both maintain established methylation and complete DNA methylation programming, even during epididymal maturation. This dynamic epigenetic landscape may confer increased vulnerability to environmental influences, including the obesogenic environment, that could disrupt reprogramming fidelity. Here we conducted an exploratory analysis that showed that overweight/obesity (*n* = 20) is associated with differences in mature spermatozoa DNA methylation profiles relative to controls with normal BMI (*n* = 47).

**Results:**

We identified 3264 CpG sites in human sperm that are significantly associated with BMI (*p* < 0.05) using Infinium HumanMethylation450 BeadChips. These CpG sites were significantly overrepresented among genes involved in transcriptional regulation and misregulation in cancer, nervous system development, and stem cell pluripotency. Analysis of individual sperm using bisulfite sequencing of cloned alleles revealed that the methylation differences are present in a subset of sperm rather than being randomly distributed across all sperm.

**Conclusions:**

Male obesity is associated with altered sperm DNA methylation profiles that appear to affect reprogramming fidelity in a subset of sperm, suggestive of an influence on the spermatogonia. Further work is required to determine the potential heritability of these DNA methylation alterations. If heritable, these changes have the potential to impede normal development.

## Background

A growing body of evidence supports that early-life environmental exposures can increase the risk of adult chronic disease. Effects of environmental exposures may be mediated through epigenetic changes, including changes in DNA methylation [1]. The patterns of DNA methylation throughout the genome (referred to as the methylome) help to regulate temporal and spatial gene expression. Plasticity of the methylome lends itself to heightened vulnerability to potential detrimental errors during periods of epigenetic flux, especially during the methylation reprogramming events that take place immediately post-fertilization and during gametogenesis [2].

After puberty, sperm production is continuous throughout adult life. This requires that sperm-specific DNA methylation profiles established during reprogramming of the primordial germ cells are maintained in the maturing sperm cells, and final methylation patterns must be established. The DNA methyltransferase enzymes are present throughout spermatogenesis, and studies in rodents have shown that de novo DNA methylation is continued even after the spermatids transit into the epididymis for maturation [3, 4]. As a result of this continual need for methylation maintenance and completion of reprogramming, DNA methylation in male gametes may be more vulnerable to exogenous and endogenous environmental influences, including an obesogenic environment [5–11].

We previously demonstrated that babies born to obese fathers have altered DNA methylation at several regulatory regions of imprinted genes [10, 11]. Imprinted genes are defined by DNA methylation that is divergent at the same genomic locations in sperm versus oocytes. These imprinted regions are differentially established after sex determination in the embryo during gametogenesis and give rise to monoallelic gene expression. Here, the active and silenced alleles in each somatic cell are determined by the epigenetic marks that distinguish the two parental copies. The ~ 100 known imprinted genes are critical mediators of early growth and development, yet they comprise a relatively small subset of the genes throughout the genome. A follow-up study sought to understand these methylation changes at imprinted regions by analyzing methylation patterns in mature spermatozoa and semen parameters of normal weight men versus men who were overweight or obese [12]. We reported significantly altered DNA methylation in sperm of the overweight and obese men as compared to the normal weight men at multiple imprinted gene regulatory regions. Herein we greatly expand our initial studies by conducting an exploratory examination of the influence of overweight/obesity on DNA methylation throughout the genome.

## Results

### Study subjects

Study subject characteristics by BMI category are presented in Table 1. Twenty of the 67 men were categorized as overweight/obese (BMI > 25), representing 29.9% of our study population. One man was excluded from the study because of a BMI of 59 kg/m^2^, and one man was excluded due to insufficient sample availability. There was no significant difference between BMI categories in education, biological paternity, sperm concentration, or sperm motility. There were significant differences between BMI categories for age, marital status, and being a patient at the Duke Fertility Center, with overweight/obese men being older, more likely to be married, and more likely to be patients. The majority of men in both BMI categories had not previously biologically fathered children. Using the Kendall’s rank correlation, we found no significant relationships between any of the semen parameters analyzed and BMI of all study subjects, nor when study subjects were stratified by BMI (overweight/obese or normal).

**Table 1:** Study participant table

	Normal weight *n* = 47*		Overweight/obese *n* = 20*		p**
	*n*	%	*n*	%	
*Age*					**0.0033**
18–24	26	55.3	3	15.0	
25–29	12	25.5	6	30.0	
30–37	9	19.1	11	55.0	
*Highest degree of education*					0.97
High school	6	12.8	1	5.0	
Some college or college degree	28	59.6	13	65.0	
Graduate	12	25.5	6	30.0	
*Marital status*					**0.0070**
Single	31	65.9	6	30.0	
Married/living with partner	16	34.0	14	70.0	
*Biologically fathered children*					0.30
No	42	89.4	16	80.0	
Yes	5	10.6	4	20.0	
*Sperm concentration*					0.78
< 15 × 10^6^/ml	3	6.4	1	5.0	
≥ 15 × 10^6^/ml	41	87.2	19	95.0	
*Sperm motility*					0.39
< 40%	7	14.9	5	25.0	
≥ 40%	37	78.7	15	75.0	
*Patient at fertility clinic*					**0.0007**
No	40	85.1	9	45.0	
Yes	7	14.9	11	55.0	

Bolded values were deemed significant with* p* < 0.05

^*^Sums less than the total reported n indicate missing data; percentage was calculated on known data

^**^p-values calculated using Chi-squared tests

### Altered DNA methylation in sperm from men with elevated BMI

The Illumina Infinium HumanMethylation450 BeadChip (hereafter, 450K) is designed to generate quantitative DNA methylation data for 485,512 CpG sites throughout the genome. In order to account for the two different probe design types on the 450K BeadChip, the CpG sites were subjected to subset quantile within-array normalization (SWAN). Dropping CpG probes with poor detection p-values resulted in retention of 485,498 probes. All unreliable probes based on prior criteria [13] were removed, reducing the number of retained probes to 294,833. Finally, probes that were invariant across all samples (*β* variance < 2 × 10^–5^) were discarded, resulting in a total of 291,061 retained CpG sites for analysis [14, 15, 17].

**Fig. 1:**
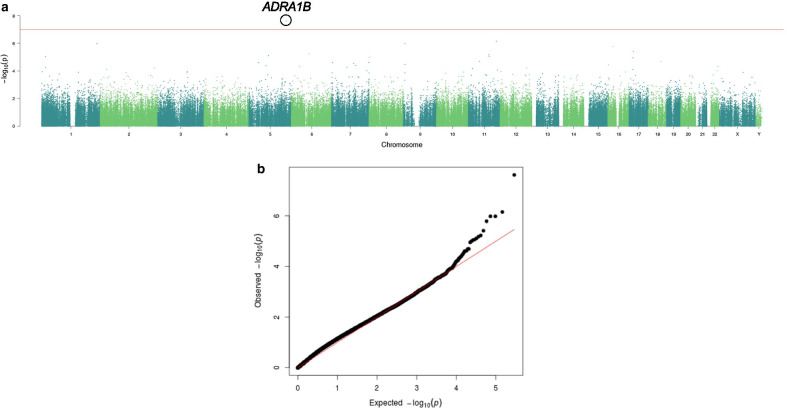
Methylation differences across the genome between sperm of men with normal versus overweight/obese BMIs. **a** Manhattan plot showing the distribution of significance levels [*y* axis, − log_10_(*p*)] across the genome, by genomic coordinates along each chromosome (*x* axis). **b** Quantile–quantile plot showing the distribution of expected *p* values (− log_10_(*p*); *x* axis) plotted against observed *p* values (*y* axis)

Linear regression analysis was conducted on the 291,061 CpG sites controlling for age, race, smoking status and clinic patient status. A higher BMI was associated with one CpG site, cg24769403, which passed significance at the false discovery rate (FDR *p* = 0.007; 9.9% higher methylation in overweight/obese men) and the more conservative Bonferroni correction (*p* = 1.0 × 10^–7^; Fig. 1). This CpG site lies 70.9-kb downstream of the transcription start site of the protooncogene, *ADRA1B*. None of the other CpGs were significant after adjustment for multiple comparisons. We therefore analyzed the data based on the raw *p* values (*p* < 0.01), which showed there were 3,264 CpG sites significantly associated with BMI, with *p* values ranging from *p* = 0.01 to *p* = 2.4 × 10^–8^ (average and median *p* = 5.3 × 10^–3^). The FDR *p* values for all CpG sites can be found in Additional file 2: Table S1. Of these, 2,851 were associated with unique gene names. The probes associated with the top 20 differentially methylated CpGs are shown in Table 2. There were 315 genes with multiple significant CpG sites per gene (range 2–19). The top two genes with multiple affected CpGs were Protein Tyrosine Phosphatase Receptor Type N2 (*PTPRN2*; 19 sites with methylation values ranging from 3.4% lower to 3.6% higher in healthy versus overweight/obese; unadjusted p values ranging from 2.4e−5 to 0.007) and zinc finger protein 33A (*ZNF33A*; 10 sites with methylation values ranging from 1.3% to 6.4% lower in healthy versus overweight/obese men; unadjusted p values ranging from 0.0001 to 0.008).

**Table 2:** Top 20 differentially methylated CpG probes

CG probe ID	Nearest UCSC RefGene name	Chr	Avg *β* normal weight	Avg *β* overweight obese	Unadjusted *p*	Adjusted *p*
cg24769403	*ADRA1B*	5	0.021	0.119	2.43E−08	0.007
cg19350020	*PHLDB1*	11	0.930	0.883	7.09E−07	0.077
cg13985597	*RP11-390F4.10*	9	0.053	0.077	1.06E−06	0.077
cg10578952	*TBCE*	1	0.935	0.919	1.06E−06	0.077
cg07425780	*GPRC5B*	16	0.056	0.080	1.64E−06	0.096
cg04863514	*FLCN*	17	0.920	0.898	3.83E−06	0.186
cg14375912	*COL12A1*	6	0.051	0.067	5.93E−06	0.239
cg04147990	*PRSS23*	11	0.911	0.881	6.59E−06	0.239
cg02371408	*VCAN*	5	0.070	0.089	7.72E−06	0.239
cg02647408	*GRM5*	11	0.067	0.058	8.58E−06	0.239
cg16491274	*FBXO42*	1	0.032	0.043	9.03E−06	0.239
cg00931944	*AF067845.1*	8	0.846	0.812	9.97E−06	0.242
cg06960881	*PMP22*	17	0.867	0.878	1.11E−05	0.249
cg23072973	*C11orf49*	11	0.817	0.800	2.01E−05	0.395
cg15444472	*CTD-2526M8.3*	18	0.900	0.909	2.04E−05	0.395
cg18566911	*PTPRN2*	7	0.885	0.877	2.38E−05	0.403
cg15319585	*SDK1*	7	0.897	0.914	2.43E−05	0.403
cg11670605	*PLCXD3*	5	0.019	0.016	2.49E−05	0.403
cg04712949	*CALCR*	7	0.061	0.056	2.90E−05	0.445
cg13762612	*RP11-390F4.10*	9	0.078	0.110	3.30E−05	0.480

To determine whether there were functionally related groups of genes that were targeted by BMI-associated alterations in sperm DNA methylation, we entered the top 3000 gene names associated with the lowest *p* values (*p* value range, 2.4 × 10^–8^ to 9.0 × 10^–3^) into the DAVID Bioinformatics Database 6.8 [17, 18]. Using the default *Homo sapiens* population of background genes provided by DAVID, results indicated significant enrichment of Biological Process Gene Ontology (GO) terms, including GO:0045944 and GO:0000122, “positive” and “negative regulation,” respectively, “of transcription from RNA polymerase II promoters” (176 genes, *p* = 1.2 × 10^–12^; and 132 genes, *p* = 2.7 × 10^–10^, respectively) and GO:0007399, “nervous system development” (65 genes, *p* = 5.3 × 10^–9^) as the top three. Others included GO:0007411, “axon guidance” (38 genes; *p* = 3.0 × 10^–6^) and GO:0007416, “synapse assembly” (20 genes; *p* = 1.0 × 10^–5^). (Table 3). Analysis of KEGG pathways showed enrichment of hsa04550, “signaling pathways regulating pluripotency of stem cells” (30 genes, *p* = 1.8 × 10^–4^), and hsa05202, “transcriptional misregulation in cancer” (33 genes, *p* = 3.9 × 10^–4^) (Table 4).

**Table 3:** Significant GO terms

GO category	Pathway	*p* value	Benjamini value
GOTERM_BP_DIRECT	Positive regulation of transcription from RNA polymerase II promoter	1.24E−12	6.32E−9
GOTERM_BP_DIRECT	Negative regulation of transcription from RNA polymerase II promoter	2.65E−10	6.78E−7
GOTERM_BP_DIRECT	Nervous system development	5.27E−9	8.99E−6
GOTERM_BP_DIRECT	Positive regulation of transcription, DNA-Templated	1.02E−8	1.30E−5
GOTERM_BP_DIRECT	Inner Ear Morphogenesis	1.27E−7	1.3E−4
GOTERM_BP_DIRECT	Transcription from RNA polymerase II promoter	1.74E−6	1.45E−3
GOTERM_BP_DIRECT	Axon guidance	3.01E−6	2.19E−3
GOTERM_BP_DIRECT	Thymus development	3.83E−6	2.45E−3
GOTERM_BP_DIRECT	Transcription, DNA-Templated	4.83E−6	2.74E−3
GOTERM_BP_DIRECT	Synapse assembly	1.02E−5	5.19E−3
GOTERM_BP_DIRECT	Embryonic cranial skeleton morphogenesis	3.96E−5	1.82E−2
GOTERM_BP_DIRECT	Cell migration involved in sprouting angiogenesis	5.07E−5	2.1E−2
GOTERM_BP_DIRECT	Embryonic forelimb morphogenesis	5.7E−5	2.2E−2
GOTERM_BP_DIRECT	Hemopoiesis	8.8E−5	3.2E−2
GOTERM_BP_DIRECT	Thyroid gland development	1.3E−4	4.2E−2
GOTERM_BP_DIRECT	Pancreas development	1.5E−4	4.6E−2

**Table 4:** Significant KEGG pathways

KEGG category	TERM	*p* value	Benjamini value
KEGG_PATHWAY	hsa04559:Signaling pathways regulating pluripotency of stem cells	1.8E−4	5.1E−2
KEGG_PATHWAY	hsa05202:Transcriptional misregulation in cancer	3.9E−4	5.4E−2
KEGG_PATHWAY	Hsa04925:Aldosterone synthesis and secretion	4.68E−4	4.3E−2

### Genomic imprinting results

The current comprehensive approach is also consistent with our earlier findings of imprinted genes in the same study population. We earlier reported that obesity was associated with lower methylation at the *MEG3*, *SNRPN*, and *SGCE/PEG10* DMRs, and increased DNA methylation at *MEG3-IG* and *H19* DMRs [12]. We observed overlap in significance and direction of methylation change at all of these regions except *MEG3-IG*, which is not included on the 450K platform (*MEG3*: cg23870378, 1.0% lower, *p* = 0.01; cg05711886: 1.1% lower, *p* = 0.01; *SNRPN*: cg21870668: 0.98% lower, *p* = 0.007; cg02152271, 2.0% lower, *p* = 0.02; *SGCE* (intron 1): cg21743410, 0.82% lower, *p* = 0.03; cg20528183, 0.24% lower, *p* = 0.04; *PEG10* (intron 1): cg05509218, 1.1% lower, *p* = 0.01; and cg22820921, 0.49% lower, *p* = 0.01). Notably, the latter probe CG site is included within the exact region identified as exhibiting decreased methylation in the overweight/obese from our prior study [12]. We also observed increased methylation for *H19* (cg15963714: 1.4% higher, *p* = 0.007). There is no overlap in the other regions identified here using the 450K platform and the specific CG sites analyzed in our prior analysis.

### Pyrosequencing results

**Fig. 2:**
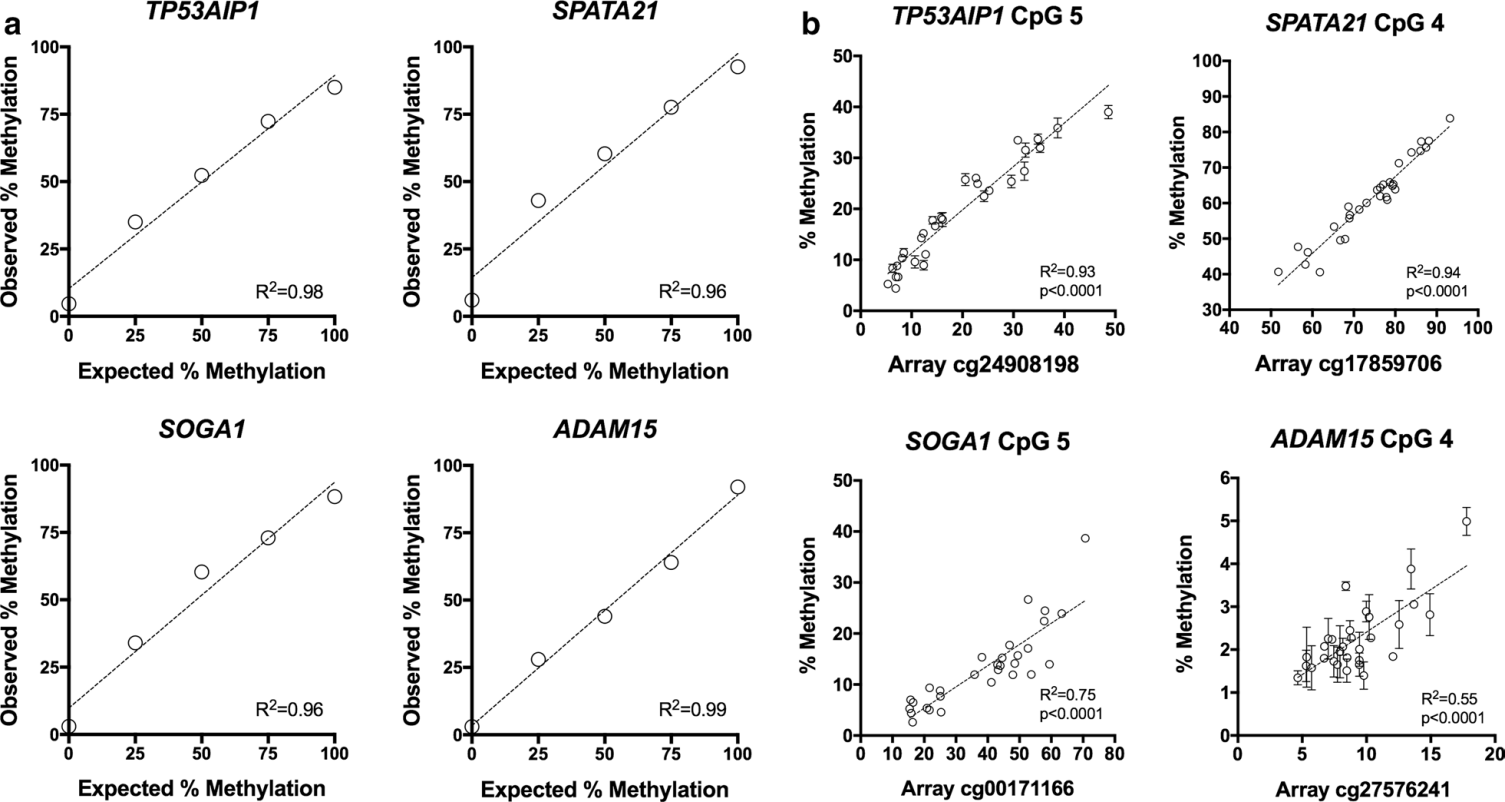
Validation of select methylation values obtained on the Illumina HumanMethylation450 BeadChip using an independent quantitative method. **a** Confirmation of pyrosequencing assay performance whereby methylation input (*x* axis) was compared to measured methylation (*y* axis) using defined mixtures of fully methylated and unmethylated DNAs. Data shown are the mean of triplicate measures. Some standard  deviations  were too small to be visible on the graph. **b** Comparison of DNA methylation measured on the Illumina platform (*x* axis) versus that measured by pyrosequencing (*y* axis) for the same CpG sites for *n* = 30 individuals. The average of duplicate measures is shown ± SD

To confirm results from our 450K data, we performed bisulfite pyrosequencing of all remaining sperm DNA samples for target regions of arbitrarily chosen genes (Table 5) that were designed to measure methylation at the identified differentially methylated CpG site from the 450K platform, including Tumor protein P53 regulated apoptosis-inducing protein 1 (*TP53AIP1*), spermatogenesis-associated 21 (*SPATA21*), suppressor of glucose, autophagy associated 1 (*SOGA1*), and ADAM metallopeptidase domain 15 (*ADAM15*). Figure 2a shows the results for pyrosequencing assay performance, where input methylation using defined mixtures of fully methylated and unmethylated bisulfite converted control DNAs agreed with that measured by pyrosequencing for all four assays (Pearson *R*^2^ = 0.96 to *R*^2^ = 0.99).

**Table 5:** CpG sites selected for validation

CG site	UCSC RefGene name	CHR	Avg *β* value overweight/obese	Avg *β* value normal weight	Difference in *β*	No. of associated CG sites	*p* value
cg24908198	*TP53AIP1*	11	0.1823	0.2331	0.0508	2	< 0.01
cg17859706	*SPATA21*	1	0.5510	0.6140	0.0630	1	< 0.01
cg00171166	*SOGA1*	20	0.3411	0.3918	0.0507	1	< 0.01
cg27576241	*ADAM15*	1	0.1082	0.1334	0.0252	1	< 0.01
cg05772935	*MAPK8IP3*	16	0.7174	0.6112	0.1143	4	< 0.01
cg17169982	*TBCD*	17	0.2941	0.3210	0.0269	1	< 0.01
cg25398727	*XKR6*	8	0.7814	0.6950	− 0.0864	2	< 0.01
cg18870054	*MISP*	19	0.5356	0.4688	− 0.0668	3	< 0.01
cg01098939	*AMZ1*	7	0.8160	0.9008	0.0848	1	< 0.001
cg18578876	*HCAR3*	12	0.7496	0.8654	0.1158	1	< 0.01

**Fig. 3:**
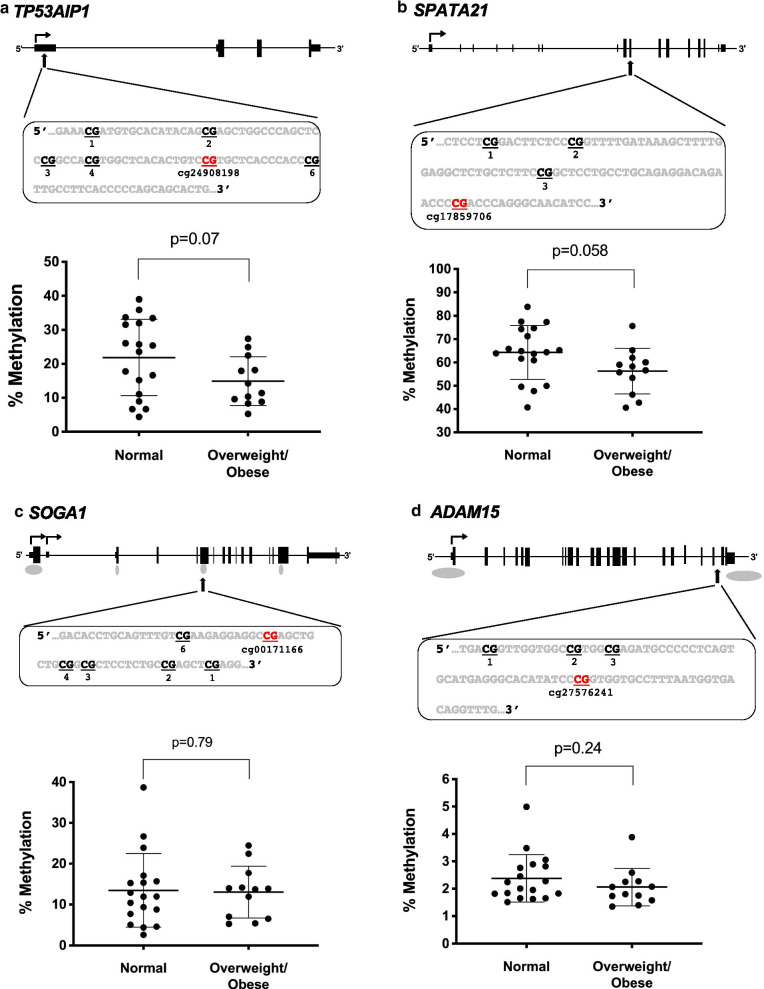
Pyrosequencing of candidate CpG sites, comparing values obtained from sperm of men with normal BMIs to those with overweight/obese BMIs. **a** Pyrosequencing data show differences between men with normal BMI (*n* = 18) and overweight/obese men (*n* = 12) for **a** *TP53AIP1* (unpaired t test), **b** *SPATA21* (unpaired t test) but not **c** *SOGA1* (Mann–Whitney test) or **d** *ADAM15* (Mann–Whitney test)*.* The corresponding gene schematics with the sequence to analyze are above each gene, with the CpG site identified via 450K highlighted in red and the probe ID included

Figure 2b shows the sperm DNA methylation levels measured by pyrosequencing directly compared to the sperm DNA methylation levels measured on the 450K platform for 30 subjects. The degree of methylation at each specific CpG site measured by pyrosequencing correlated with that of the methylation measured on the 450K platform for all genes (Pearson *R*^2^ = 0.93, *p* < 0.0001 for *TP53AIP1*; *R*^2^ = 0.94, *p* < 0.0001 for *SPATA21*; *R*^2^ = 0.75, *p* < 0.0001 for *SOGA1*; *R*^2^ = 0.55, *p* < 0.0001 for *ADAM15*; data from all analyzed sites for each gene are provided in Additional file 3: Table S2). Although the overall levels of methylation for *ADAM15* were very low, we nevertheless were able to detect a significant positive correlation between pyrosequencing and 450K values. We then examined the methylation levels at all four differentially methylated CpG sites of the overweight/obese versus normal weight men with available remaining sample. The sperm of normal weight men had higher DNA methylation levels than sperm of overweight/obese men for *TP53AIP1* (21.9% ± 2.7 vs 14.9% ± 2.1, p = 0.07) and in normal weight compared to overweight/obese men for *SPATA21* (64.3% ± 2.7 vs 56.3% ± 2.8, *p* = 0.058) which was in accordance with our results from the 450K platform (Fig. 3a, b). *SOGA1* showed no difference in methylation between the groups by pyrosequencing (*p* = 0.79), and while results for *ADAM15* were not significant (*p* = 0.24), they were in agreement with that observed on the 450K platform with respect to men with normal weight having higher methylation than those who were overweight/obese (Fig. 3c and d; data for all CpG sites analyzed are shown in Additional file 1: Figure S2).

### Cloned allele sequencing results

Since the methylation changes that were measured using the 450K and the pyrosequencing data are both representative of the averaged sperm population as a whole in the sample analyzed, we wanted to determine whether the differences in DNA methylation were evident by analyzing differentially methylated CpG sites in multiple single sperm from the same individual. By so doing, we can assess whether the methylation changes occur in a manner that is randomly distributed across all sperm or whether these changes only affect a small subset of sperm. We used bisulfite sequencing of cloned alleles to address this, whereby each individual clone reveals the methylation status of every CpG within the contiguous sequenced region of a single sperm cell, and multiple cloned alleles were sequenced for each individual analyzed. We selected seven regions for analysis (Table 5) that showed the largest methylation differences between normal weight and overweight/obese men. The participants showing the most divergent results from the 450K platform analysis and with remaining sample were chosen for the cloned allele studies. For three genes, mitotic spindle positioning (*MISP*), archaelysin family metallopeptidase 1 (*AMZ1*), and hydroxycarboxylic acid receptor 3 (*HCAR3*), there were no major differences between the normal weight and overweight/obese sperm samples in terms of the distribution of methylation across the individual alleles (Additional file 1: Figure S1).

**Fig. 4:**
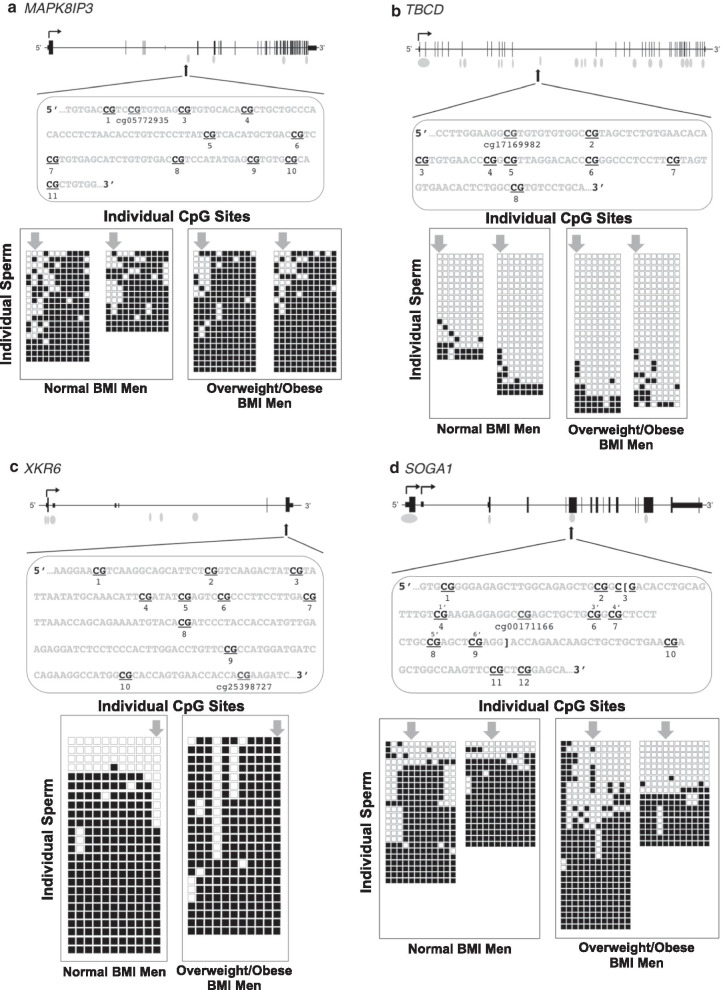
Non-random distribution of methylation changes across the sperm population by bisulfite sequencing of cloned alleles. For each gene, the genomic structure and relative position of the region sequenced are shown, with the actual sequence of the region, and CpG sites queried shown below. The CpG that exhibited differential methylation on the Illumina HumanMethylation450 (450K) bead chip is indicated, along with the probe ID. The numbering of the CpGs below the sequence corresponds to each of the CpGs analyzed. For *SOGA1*, the bracketed sequence and the numbering of CpGs from 1′ to 6′ are the CpG sites analyzed by bisulfite pyrosequencing (refer to Figs. 2 and 3). For each region, the PCR products derived from bisulfite-modified sperm DNA were cloned and sequenced from either two (panel **c**) or four (panels **a**, **b** and **d**) individuals per region. Results from men with a normal BMI are shown on the left for each gene and men with an overweight/obese BMI are shown on the right. The results for each individual are represented by a tight grouping of boxes, with the columns representing each CpG position in the sequence shown above, with numbering of each CpG from left to right. The rows represent the results for one individual clone. For example, in Panel **a**, data are shown for two men with normal BMI and two with overweight/obese BMI. There are 11 CpG sites analyzed for each individual, with 19 and 14 alleles, respectively, shown for the two men with normal BMI and 21 alleles each shown for the men with overweight/obese BMI. Filled boxes indicate the CpG is methylated; unfilled boxes indicate the CpG is unmethylated. The arrows point to the individual CpG detected as differentially methylated on the 450K platform

However, the other four genes analyzed showed differences in methylation profiles between the sperm cells within a given individual as well as differences between normal weight and overweight/obese individuals. For mitogen-activated protein kinase 8 interacting protein 3 (*MAPK8IP3)*, there were fewer methylated CpG sites across the sperm analyzed in the normal weight (average 76.7% methylation) compared to the overweight/obese sperm samples (average 87.7% methylation) (Fig. 4a). For tubulin-folding cofactor D (*TBCD*), the sperm of all men analyzed were mostly unmethylated except for several from each individual with a more heavily methylated profile (Fig. 4b). For XK related 6 (*XKR6*), 16% of the sperm were nearly completely unmethylated, while the remainder were highly or fully methylated in normal weight men (Fig. 4c). In the overweight/obese sample, all of the sperm were heavily methylated (88.5%). Finally, for *SOGA1*, the majority of the sperm were heavily methylated in the normal weight men, whereas there were a roughly equal number of heavily methylated and largely unmethylated sperm in the overweight/obese men (Fig. 4d).

## Discussion

Approximately 34% of adult males are classified as obese in the USA [19], and obesity is one of the major contributors to male-factor infertility [20]. This relationship is likely driven through increased energy input with consequent inflammation, disruption of metabolism and endocrine signaling [21]. Thus, as we have previously suggested [22], changes in the molecular composition of sperm can impact DNA methylation. As such, we sought to expand upon our prior study of imprinted gene regulatory regions [12] to determine whether there were detectable alterations elsewhere in the genome.

We found significant differences in DNA methylation comparing sperm from overweight/obese men to normal weight men at multiple CpG sites. From our exploratory 450K dataset, there was one CpG site that was significant at the FDR that is located downstream of the adrenoreceptor alpha 1B gene (*ADRA1B). ADRA1B* is a protooncogene that is a member of the alpha-1-adrenergic receptor family. This family of receptors activates mitogenic responses and plays a role in the regulation of cellular growth and proliferation. In particular, *ADRA1B,* when transfected into NIH 3T3 fibroblasts, induces the neoplastic transformation of cells. Future studies might focus on how lifestyle factors and environmental exposure might impact DNA methylation in additional regions of this gene. Many of the identified genes have key regulatory roles in developmental, metabolic, and inflammatory processes. As such, alterations due to DNA methylation changes could have significant downstream effects. A large number of the gene ontology and KEGG terms associated with the identified differentially methylated sites are related to early embryonic and neuronal development, and the regulation (or misregulation) of transcription. Genes critical for early embryonic and neuronal development, as well as the regulation of transcription, are among the genes that are poised for post-fertilization activation in sperm, given their critical function during early-life development. Their required early activation of expression, however, appears to make them more susceptible to environmental perturbations that can disrupt their proper methylation. If changes in DNA methylation at these genes are retained post-fertilization, this could lead to potential unintended consequences during development due to dysregulated expression.

From the 3264 differentially methylated CpG sites that distinguish normal weight from overweight/obese men (unadjusted *p* value < 0.01), we arbitrarily chose sites for validation. The DNA methylation values measured by bisulfite pyrosequencing for CpG sites associated with *TP53AIP1*, *SPATA21*, *SOGA1* and *ADAM15* were highly correlated with the values obtained on the 450K platform. *TP53AIP1* encodes a TP53-inducible protein involved in mediating apoptosis [23]. SPATA21 is involved in the differentiation of haploid spermatids. A gene-based association study has shown that *SPATA21* is one of several genes implicated in adolescent idiopathic scoliosis [24]. SOGA1 regulates autophagy by playing a role in reducing glucose production in an adiponectin-mediated and insulin-dependent manner [25]. Finally, *ADAM15* is a protein coding gene that is a member of the ADAM (a disintegrin and metalloproteinase) protein family which are transmembrane glycoproteins involved in cell adhesion. This protein family is thought to play diverse roles in cellular processes, one of which includes fertilization and, in fact, is among candidates that may be the binding entities at the egg membrane surface [26]. In guinea pig spermatozoa, ADAM15 interacts with the cell adhesion glycoprotein acrogranin during the fertilization process [27].

The findings of our study are consistent with the results of studies by Donkin et al. [28] as well as Potabattula et al. [29], where the potential effects of obesity on the sperm epigenome were investigated, with a focus on potential intergenerational inheritance in the latter. Donkin et al. identified 9081 unique differentially methylated genes between 13 normal weight and 10 obese men. In addition, they analyzed sperm from six obese men before and after bariatric surgery. They found that a large number of genes in sperm showed changes in DNA methylation a week after surgery and that these new profiles were maintained in the sperm for at least a year. Such rapid changes in sperm DNA methylation suggest that the alterations were induced in the maturing sperm, since the timeframe from spermatogonial differentiation to production of mature sperm is about 74 days in humans. That the alterations were detectable one year later may be indicative of a simultaneous and permanent methylation change in the spermatogonial progenitors. Potabattula et al. [29] examined DNA methylation by bisulfite pyrosequencing at seven imprinted genes and one non-imprinted gene in sperm of normal weight men, pre-obesity/obese men, and one underweight man, as well as in the cord blood of offspring. The researchers found a positive correlation at the *MEG3-*IG regulatory region between sperm DNA methylation and BMI. They also reported a sex-specific correlation between paternal BMI and methylation levels in cord blood for the *MEG3-*IG DMR, *IGF2-*DMR0, and *HIF3A*, the non-imprinted gene the group analyzed. Additionally, hypomethylation of *IGF2-*DMR0 in fetal cord blood was associated with increased paternal BMI in female offspring. These results support our findings that there are detectable BMI-related differences in sperm DNA methylation and support that these altered methylation patterns can be passed onto offspring.

The current analysis is consistent with our prior findings on genomic imprinting for the regions that were included on the 450K platform. We were able to compare the CpG sites that are represented on the 450K platform with what we had previously published and found agreement with what we had observed in our prior work. Further, the concordance between genes and direction of methylation change between the 450K platform used here and the pyrosequencing data from our prior study support the validity of our findings.

It has generally been thought that the reprogramming events that occur during gametogenesis and post-fertilization leave little chance to transmit any altered methylation profiles from the prior generation to the next. Gametic epigenetic reprogramming has been thought of as evolution’s way to ensure undoing of any potentially harmful changes that may have occurred during a parent’s lifetime [30]. On the other hand, environmentally induced epigenetic changes in gametes could be transferred to subsequent generations, which might explain how relatively fast evolutionary responses result from environmental changes [9, 31–33]. Recent studies have shown that a substantial number of regions of the genome are resistant to the DNA methylation erasure that occurs during gametogenesis and post-fertilization reprogramming [34–36]. The partial retention of DNA methylation at these “escapee” regions may provide a way of transmitting intact epigenetic information to the next generation.

The haploid nature of sperm cells makes it possible to use bisulfite sequencing of cloned alleles as a method to examine the patterns of DNA methylation present in individual sperm cells and the distribution of these patterns across multiple sperm from the same individual. Apart from the potential limitation of selecting multiple clones representing the same sperm, our analysis suggests that CpG methylation alterations in sperm at the regions analyzed are not randomly distributed throughout the entirety of the sperm population, but rather appear to be present in a small proportion of the sperm cells. Furthermore, the differences in methylation associated with an overweight/obese BMI are reflected by a shift in these proportions. From our results, we are unable to determine whether the same sperm cell is impacted by methylation changes at more than one of these regions. Nevertheless, these results indicate that overweight/obese men have an increased chance of conceiving a child with sperm carrying a skewed methylation configuration at one or more regions of the genome.

Study limitations include the exploratory nature of the study and a small sample size with recruitment of one-third of our study population from the Duke Fertility Center. We adjusted for Fertility Center patient status and excluded males with known male factor infertility, but it is possible that there is residual confounding due to inherent differences in characteristics of sperm DNA between men attending the clinic and those not attending the clinic. We also limited our study population to Caucasian men due to potential differences in DNA methylation based on race/ethnicity. One of the reasons for potential lack of validation by cloned allele analysis for some regions is that this methodology examines only a small proportion of the total sperm population, and there can be bias in PCR amplification as well as in selection of individual clones for sequencing. Nonetheless, some of our data from this analysis suggest that we were able to detect differences in the distribution of methylation for a number of the genes examined. Finally, there are more than 28 million CpG sites throughout the genome, and the ~ 486,000 included here may have missed detection of other important regions that are affected by overweight/obese status. Study strengths include restriction to Caucasian men, thus limiting heterogeneity and increasing the power to detect true associations, and that all sample processing and data generation were performed in parallel. We used two independent methods for confirmation of our findings, including rigorously developed assays for bisulfite pyrosequencing as well as bisulfite sequencing of a large number of cloned alleles. We were especially compelled by the successful validation of some of our targets, given that these sites were indeed chosen at random, and not because of the magnitude of their methylation difference or their degree of statistical significance. Our analysis indeed showed that there are multiple different allelic methylation profiles at the same locus in sperm from the same individual.

## Conclusions

Our study contributes to the growing body of evidence that the impact of paternal lifestyle on the proper maturation of the epigenetic information carried in the sperm, and potentially on subsequent embryonic and fetal development, is perhaps more important than previously appreciated. Obesity-related epigenetic changes in sperm may be reversible with weight loss, and thus, improving paternal metabolic health is anticipated to increase the proportion of sperm with a more healthy methylome and therefore decrease the chances of an adverse impact on embryonic and fetal development [28, 37]. Given the obesity epidemic, it is essential to replicate our results in a larger sample size. Genetics needs to also be examined, since CpG methylation can be influenced by genotype [38–40]. Lastly, our results underscore an urgent need to determine the potential for inter- and transgenerational heritability of altered sperm DNA methylation profiles.

## Methods

### Study participation and data collection

All participants in this study were enrolled in the TIEGER study at Duke University. Subject recruitment and inclusion/exclusion criteria were previously described, and subjects were excluded if they had known male factor infertility [12]. BMI categories were defined in accordance with World Health Organization guidelines as follows: normal weight (18.5 kg/m^2^ ≤ BMI ≤ 25 kg/m^2^), overweight (25 kg/m^2^ < BMI < 30 kg/m^2^) and obese (BMI ≥ 30 kg/m^2^). For the purpose of this study, subjects with BMI > 25 were categorized as “overweight/obese.” Subjects completed a short questionnaire regarding information on socio-demographic and lifestyle factors, including level of education, marital status, number of children fathered, occupation, and physical activity. Semen, urine, and blood samples were collected from all subjects. The sample collection and processing have been previously described in detail [12].

### DNA isolation and methylation analysis

Sperm genomic DNA was extracted using Puregene Reagents (Qiagen; Valencia CA). One microgram of purified DNA for each sample was provided to the Duke Molecular Genomics Core for generation of Illumina HumanMethylation450 BeadChip data according to the manufacturer’s instructions (Illumina Inc., San Diego, CA).

The array analysis of methylation levels at each CpG site generated a *β*-value, which represents the proportion of signal obtained for methylation at a specific CpG site, where 1 is completely methylated and 0 is completely unmethylated. A logit transformation was applied to the *β*-values, due to the severe heteroscedasticity of highly methylated and unmethylated *β*-values. The transformed values, or M-values, are defined as: M = log (*β*/1 − *β*). The M-values were then used to find differential methylation [41].

### Identification of differentially methylated CpG Sites

A site-based analysis was performed using linear regression which examined each CpG site and ordered the list of individual CpG sites by the association between level of methylation and BMI as continuous variables. Potential confounders were selected based on known or observed association with DNA methylation and with obesity. In the final analysis, all results were adjusted for age, smoking status, strenuous exercise (based on median), and clinic patient status. Exercise was categorized as a binary variable, including those who exercised 0–3 days per week and those who exercised 4–7 days per week. Although no current smokers were recruited for the study, six subjects reported a prior history of smoking and were considered for the purposes of covariate adjustment. Multicollinearity between covariates was tested, and no correlations of interest were found. After the significance level for the relationship between BMI and methylation was obtained for each CpG site, the p-values were adjusted to correct for false discovery rate (FDR), denoted as the q-value.

### Bisulfite pyrosequencing

Bisulfite modification of 800 ng of sperm DNA was performed using the Zymo EZ DNA Methylation Kit, converting unmethylated cytosines to uracils while leaving methylated cytosines unaltered. The DMRs associated with the following genes were examined: *TP53AIP1* (probe cg24908198; 6 CpG sites analyzed), *SPATA21* (probe cg17859706; 4 CpG sites analyzed), *SOGA1* (probe cg00171166; 6 CpG sites analyzed), and *ADAM15* (cg27576241; 4 CpG sites analyzed). Pyrosequencing assay design was performed using PSQ Assay Design Software v1.0 (Qiagen). Forward and reverse PCR primer sequences can be found in Additional file 4: Table S3. The 5′ end of one PCR primer from each pair was conjugated to biotin to allow for retention of one DNA strand through denaturation of the double-stranded amplicons and binding of the biotin-containing strand to streptavidin beads. Bisulfite-modified sperm DNA (40 ng) was then amplified in a 10 μl PCR reaction volume using the PyroMark PCR Kit (Qiagen) with 0.3 µl 25 mM MgCl_2_ and 0.24 μl each of the forward and reverse primers (10 mM). In addition, 1 μl of CoralLoad Concentrate (Qiagen) was added to each reaction in order to help visualize amplicons on an agarose gel.

Pyrosequencing assays were performed in duplicate in sequential runs (technical replicates) in CpG analysis mode on a Qiagen Pyromark Q96 MD Pyrosequencer, and the resulting percent methylation for each CpG site as well as bisulfite conversion efficiency was calculated using PyroQ CpG software v1.0 (Qiagen). The values shown represent the mean methylation for the replicate runs for the individual CpG sites that are represented on the HumanMethylation450 BeadChip platform. Validation of pyrosequencing assays was completed in triplicate using defined mixtures of unmethylated and methylated DNA (Epitect DNA; Qiagen).

### Bisulfite sequencing of cloned alleles

Bisulfite sequencing of cloned alleles was used to provide more comprehensive information on the specific patterns of methylation that are present in individual haploid sperm cells in the vicinity of, and including, the single CpG site found to differ based on the Illumina beadchip platform. Regions examined included those associated with *MISP* (probe cg18870054; 22 CpG sites analyzed), *AMZ1* (probe cg01098939; 19 CpG sites), *GPR109B/ HCAR3* (probe cg18578876; 8 CpG sites), *MAPK8IP3* (probe cg05772935; 11 CpG sites), *XKR6* (probe cg25398727; 11 CpG sites), *TBCD* (probe cg17169982; 8 CpG sites), and *SOGA1* (probe cg00171166; 12 CpG sites). Bisulfite-treated DNA (20 ng) was amplified using the HotStarTaq PCR kit and forward and reverse primers that do not anneal to CpG sites. Primer sequences and PCR conditions are provided in Additional file 5: Table S4. The PCR amplicons were resolved on a 2% agarose gel, excised, and purified using GenElute agarose spin columns (Sigma-Aldrich; St. Louis, MO). The eluted DNA was purified using Zymo gDNA Clean and Concentrator (Irvine, CA). The purified DNA was ligated and transformed into competent JM109 *E. coli* using the pGEM® T-Easy Vector System according to the manufacturer’s instructions (Promega; Madison, WI). The bacterial transformants were plated on LB/ampicillin/X-Gal plates and grown overnight at 37 °C. Individual colony-forming units were selected for each specimen and underwent whole-cell PCR using Qiagen HotStart *Taq* DNA polymerase kit with SP6 and T7 primers 5 µl of PCR product was loaded onto a 2% agarose gel to confirm band size was as expected. The remaining PCR product was purified using the Zymo gDNA Clean and Concentrator (Irvine, CA). Following purification, samples underwent PCR in preparation for BigDye Sequencing, using the BigDye Terminator v3.1 Cycle Sequencing Kit (Applied Biosystems, Austin, TX).

### Statistical analyses

Participant characteristics were calculated using a Chi-square analysis. Linear regression models were used to adjust for age, marital status, and fertility clinic patient status, which differed by BMI. When analyzing pyrosequencing data, linear regression models were used for comparing continuous data. Unpaired *t*-tests were used for two-way comparisons and, where relevant, a Welch’s *t* test and Mann–Whitney *U* test were applied. Three-way comparisons were performed using one-way analysis of variance (ANOVA) tests with Kruskal–Wallis tests where the data were not normally distributed followed by two-group comparisons using unpaired *t*-tests or Mann–Whitney *U* tests as relevant. These statistical analyses were performed using Prism 7 for Mac OS X version 7.0a (GraphPad Software; La Jolla, CA).

## Supplementary information

**Additional file 1: Figure S1**. Distribution of methylation across the sperm population by bisulfite sequencing of cloned alleles. **Figure S2**. Pyrosequencing of candidate CpGs and adjacent CpG sites, comparing values obtained from sperm of men with normal BMIs to those with overweight/obese BMIs.

**Additional file 2: Table S1**. Top 3,264 differentially methylated CpG sites in sperm by BMI from Illumina 450k analysis.

**Additional file 3: Table S2**. Comparisons between Illumina HumanMethylation450 Beadchip probe and pyrosequencing data.

**Additional file 4. Table S3**. Primer sequences and PCR conditions for bisulfite pyrosequencing analysis.

**Additional file 5: Table S4**. Primer sequences and PCR conditions for cloned allele analysis.

## Data Availability

The datasets generated and/or analyzed during the current study are available from the author upon request.

## Abbreviations

450K: Illumina Infinium HumanMethylation450 BeadChip
SWAN: Subset-quantile Within-Array Normalization
*TP53AIP1*: Tumor protein P53-regulated apoptosis-inducing protein 1
*SPATA21*: Spermatogenesis-associated 21
*SOGA1*: Suppressor of glucose, autophagy-associated 1
*ADAM15*: ADAM metallopeptidase domain 15
*MISP*: Mitotic spindle positioning
*AMZ1*: Archaelysin family metallopeptidase 1
*HCAR3*: Hydroxycarboxylic acid receptor 3
*TBCD*: Tubulin-folding cofactor D
*MAPK8IP3*: Mitogen-activated protein kinase 8 interacting protein 3
*XKR6*: XK related 6
DMRs: Differentially methylated regions
