# Supplementary material for: Male obesity impacts DNA methylation reprogramming in sperm
Source: Clin Epigenetics. 2021 Jan 25;13:17. doi: 10.1186/s13148-020-00997-0 (PMC7831195; doi:10.1186/s13148-020-00997-0)
Supplement: Supplementary file 1 — Additional file 1: Figure S1. Distribution of methylation across the sperm population by bisulfite sequencing of cloned alleles. Figure S2. Pyrosequencing of candidate CpGs and adjacent CpG sites, comparing values obtained from sperm of men with normal BMIs to those with overweight/obese BMIs. [file 13148_2020_997_MOESM1_ESM.pdf]

Figure S1

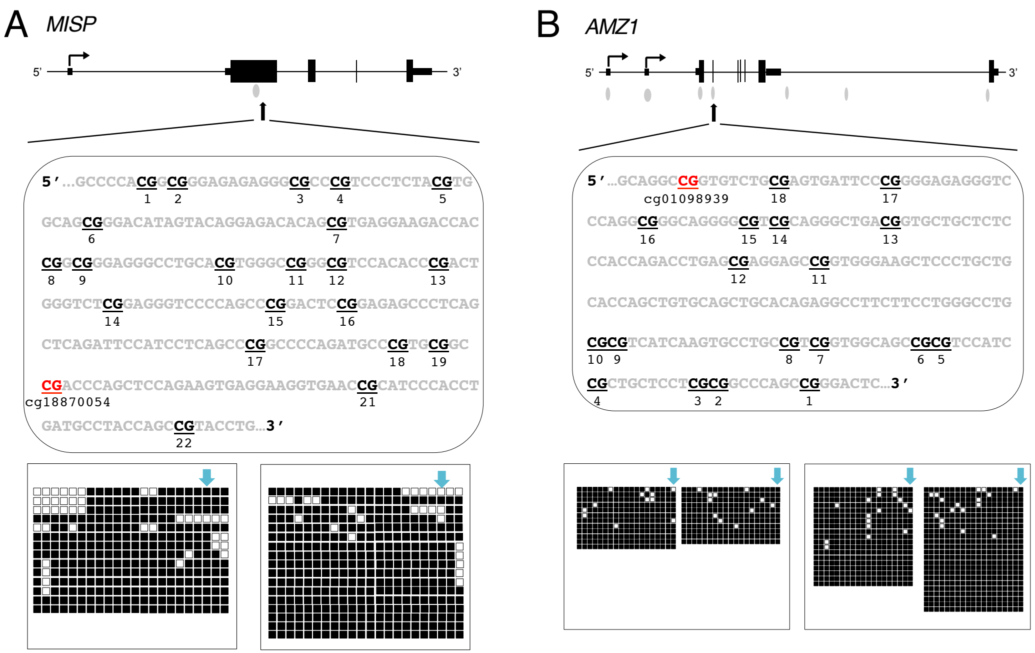

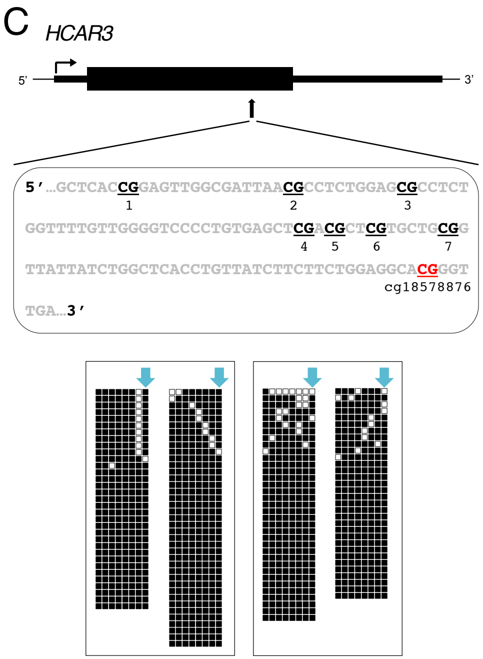

Figure S1. Distribution of methylation across the sperm population by bisulfite sequencing of cloned alleles. The genomic structure and relative position of the region analyzed is shown, with the actual sequence and CpG sites queried designated. The CpG that exhibited differential methylation on the 450k bead chip is indicated with the probe ID. The numbering of CpGs below the sequence corresponds to each of the CpGs analyzed. For each region, PCR products derived from bisulfite modified sperm DNA were cloned and sequenced from either two (panel A) or four (panels B and C) individuals per region. Results from men with a normal BMI are shown on the left for each gene and men with an overweight/obese BMI are shown on the right. The results for each individual are represented by a tight grouping of boxes, with the columns representing each CpG position in the sequence shown above, with numbering of each CpG from left to right. The rows represent the results for one individual clone. Filled boxes indicate the CpG is methylated; unfilled boxes indicate the CpG is unmethylated. The blue arrows point to the individual CpG detected as differentially methylated on the 450k platform.

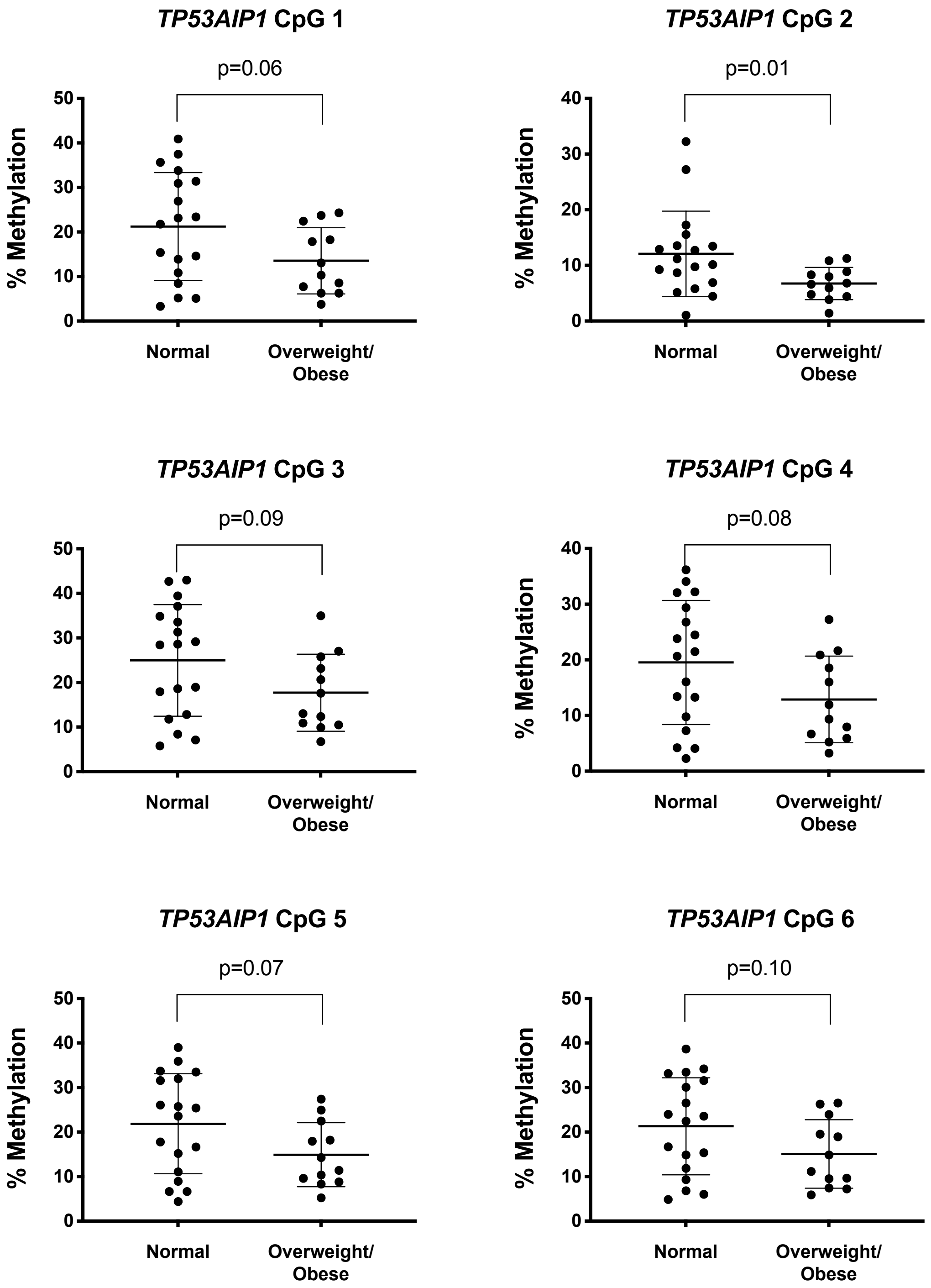

Figure S2

Pyrosequencing of candidate CpGs and adjacent CpG sites, comparing values obtained from sperm of men with normal BMIs to those with overweight/obese BMIs. Pyrosequencing data comparing men with normal BMI (n=18) to overweight/obese men (n=12) for *TP53AIP1* at the six CpG sites included in the pyrosequencing assay (see **Figure 2** for the nucleotide sequence of this region). Only CpG site 5 is represented on the Illumina Infinium Methylation450 BeadChip. Unpaired student t tests were used to compare the means at all but CpG site 2, which was analyzed using the Mann-Whitney test due to the nonnormal distribution of the data for the normal BMI group (D’Agostino & Pearson test p=0.01).

Figure S2 (cont.)

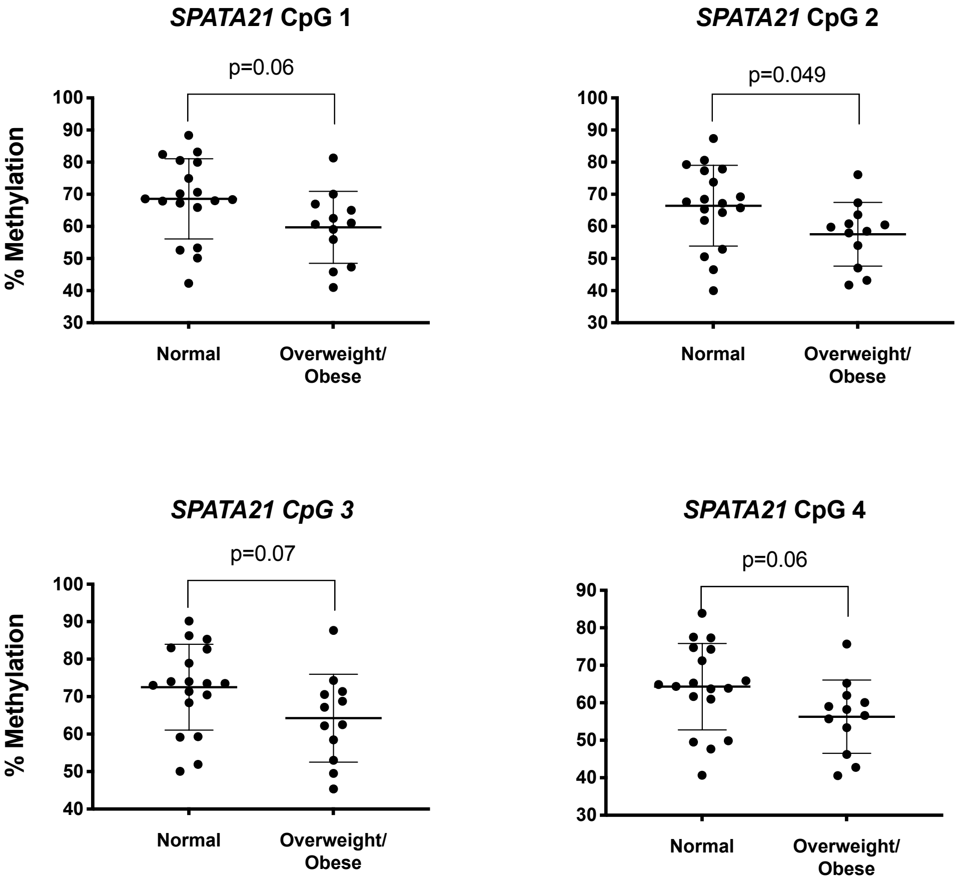

Pyrosequencing of candidate CpGs and adjacent CpG sites, comparing values obtained from sperm of men with normal BMIs to those with overweight/obese BMIs. Pyrosequencing data comparing men with normal BMI (n=18) to overweight/obese men (n=12) for *SPATA21* at the four CpG sites included in the pyrosequencing assay (see **Figure 2** for the nucleotide sequence of this region). Only CpG site 4 is represented on the Illumina Infinium Methylation450 BeadChip. Unpaired student t tests were used to compare the means.

Figure S2 (cont.)

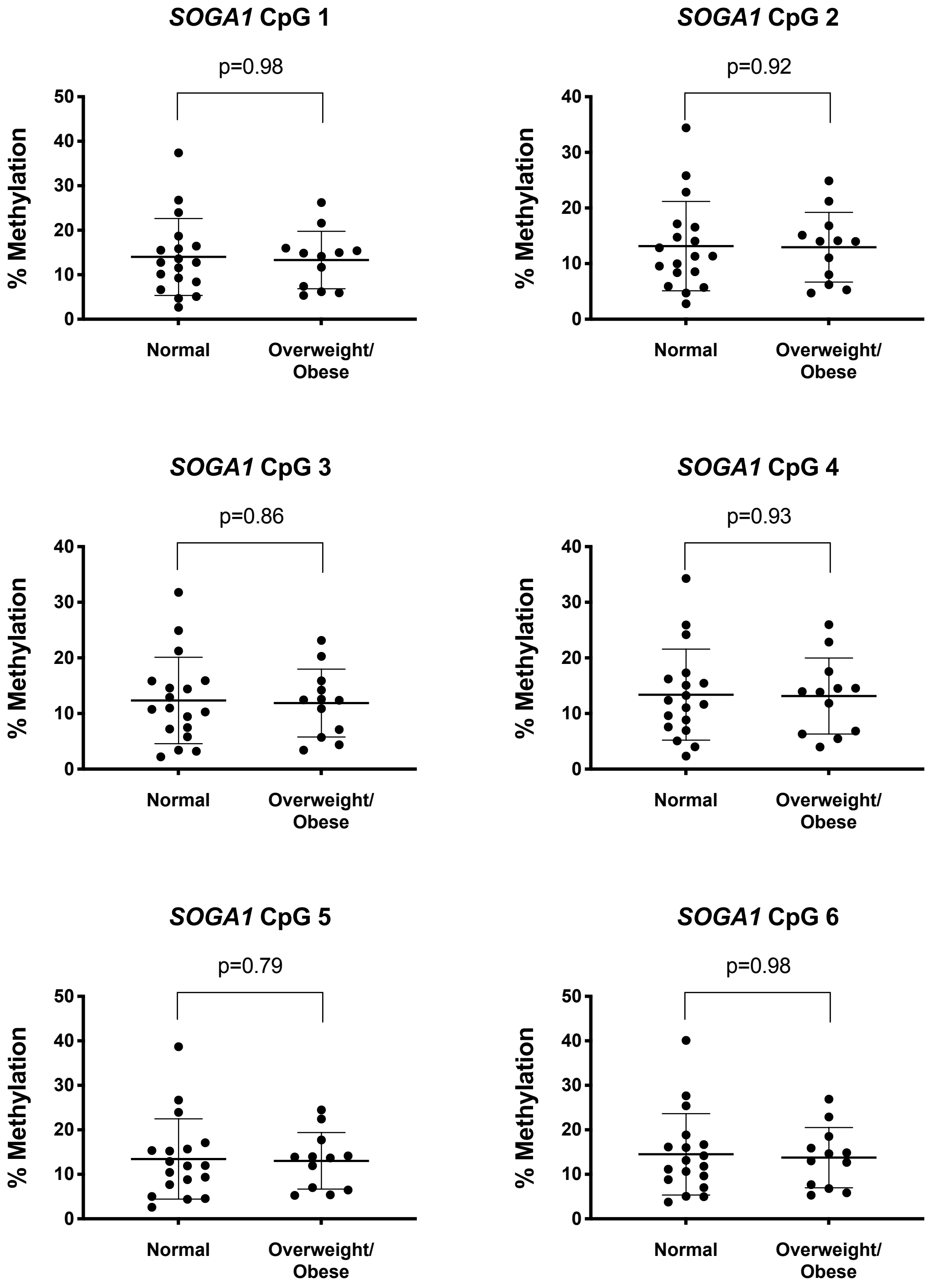

Pyrosequencing of candidate CpGs and adjacent CpG sites, comparing values obtained from sperm of men with normal BMIs to those with overweight/obese BMIs. Pyrosequencing data comparing men with normal BMI (n=18) to overweight/obese men (n=12) for *SOGA1* at the six CpG sites included in the pyrosequencing assay (see **Figure 2** for the nucleotide sequence of this region). Only CpG site 5 is represented on the Illumina Infinium Methylation450 BeadChip. Unpaired student t tests were used to compare the means at CpG sites 3 and 4, while sites 1, 2 5 and 6 were compared using the Mann Whitney test since due to the nonnormal distribution of the data for the normal BMI group at these sites (D'Agostino & Pearson test $p<0.05$).

Figure S2 (cont.)

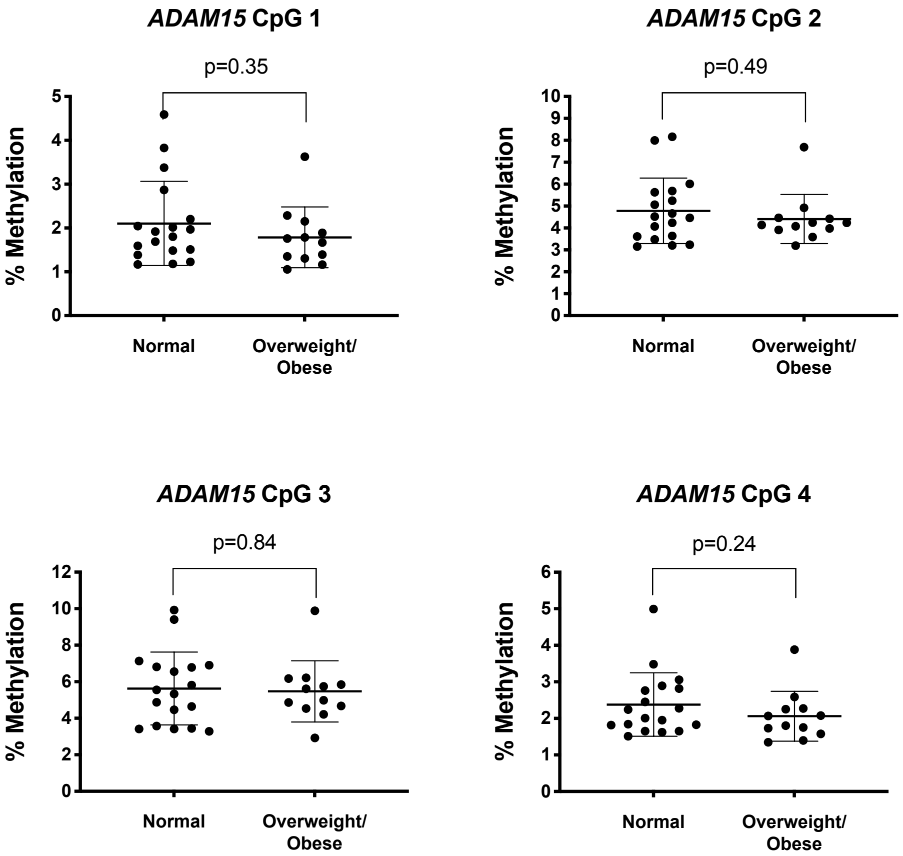

Pyrosequencing of candidate CpGs and adjacent CpG sites, comparing values obtained from sperm of men with normal BMIs to those with overweight/obese BMIs. Pyrosequencing data comparing men with normal BMI (n=18) to overweight/obese men (n=12) for *ADAM15* at the four CpG sites included in the pyrosequencing assay (see **Figure 2** for the nucleotide sequence of this region). Only CpG site 4 is represented on the Illumina Infinium Methylation450 BeadChip. All four CpG sites were analyzed using the Mann-Whitney test due to the non-normal distribution of the data for both groups for sites 1 and 4, the normal BMI group for site 2, and the overweight/obese group for site 3 (D'Agostino & Pearson test $p<0.05$).
