# Supplementary material for: Male obesity impacts DNA methylation reprogramming in sperm
Source: Clin Epigenetics. 2021 Jan 25;13:17. doi: 10.1186/s13148-020-00997-0 (PMC7831195; doi:10.1186/s13148-020-00997-0)
Supplement: Supplementary file 3 — Additional file 3: Table S2. Comparisons between Illumina HumanMethylation450 Beadchip probe and pyrosequencing data. [file 13148_2020_997_MOESM3_ESM.docx]

| **Supplemental Table 2. Comparisons between Illumina HumanMethylation450 Beadchip probe and pyrosequencing data** | | | | | | | | | | | | | | | | | | | |
| --- | --- | --- | --- | --- | --- | --- | --- | --- | --- | --- | --- | --- | --- | --- | --- | --- | --- | --- | --- |
|  | | **CpG 1** | | | **CpG 2** | | | **CpG 3** | | | **CpG 4** | | | **CpG 5** | | | **CpG 6** | | |
| **Gene** | **450k probe** | $\bar{\boldsymbol{x}}$ ^a^ **(SEM)** | **R^2^** ^b^ | **P** ^c^ | $\bar{\boldsymbol{x}}$ **(SEM)** | **R^2^** | **p** | $\bar{\boldsymbol{x}}$ **(SEM)** | **R^2^** | **p** | $\bar{\boldsymbol{x}}$ **(SEM)** | **R^2^** | **p** | $\bar{\boldsymbol{x}}$ **(SEM)** | **R^2^** | **p** | $\bar{\boldsymbol{x}}$ **(SEM)** | **R^2^** | **p** |
| ***TP53AIP1*** | cg24908198 | 18.2 (2.0) | 0.93 | <0.0001 | 9.95  (1.22) | 0.10 | 0.08 | 22.1 (2.11) | 0.92 | <0.0001 | 16.9 (1.88) | 0.91 | <0.0001 | 19.1 (1.88) | 0.93 | <0.0001 | 18.8 (1.84) | 0.92 | <0.0001 |
| ***SPATA21*** | cg17859706 | 65.0 (2.3) | 0.94 | <0.0001 | 62.9 (2.23) | 0.95 | <0.0001 | 69.2 (2.20) | 0.92 | <0.0001 | 61.1 (2.08) | 0.94 | <0.0001 |  | | | | | |
| ***SOGA1*** | cg00171166 | 13.7 (1.41) | 0.77 | <0.0001 | 13.1 (1.33) | 0.75 | <0.0001 | 12.2 (1.28) | 0.77 | <0.0001 | 13.3 (1.38) | 0.79 | <0.0001 | 13.3 * (1.45) | 0.75 | <0.0001 | 14.2 (1.49) | 0.74 | <0.0001 |
| ***ADAM15*** | cg27576241 | 1.98 (0.158) | 0.58 | <0.0001 | 4.63 (0.246) | 0.33 | 0.001 | 5.57 (0.337) | 0.34 | 0.0004 | 2.25 (0.146) | 0.55 | <0.0001 |  | | | | | |
| a, mean methylation for all samples analyzed at that CpG site with standard error of the mean (SEM)  b, r squared value for simple linear regression of methylation values obtained for the CpG probe on the HumanMethylation450 BeadChip platform and each of the CpG sites analyzed by pyrosequencing  c, p value for linear regression analysis  Green shading designates the CpG site that corresponds to the indicated 450k probe on the HumanMethylation450 BeadChip | | | | | | | | | | | | | | | | | | | |
