# Supplementary material for: Male obesity impacts DNA methylation reprogramming in sperm
Source: Clin Epigenetics. 2021 Jan 25;13:17. doi: 10.1186/s13148-020-00997-0 (PMC7831195; doi:10.1186/s13148-020-00997-0)
Supplement: Supplementary file 4 — Additional file 4. Table S3. Primer sequences and PCR conditions for bisulfite pyrosequencing analysis. [file 13148_2020_997_MOESM4_ESM.docx]

| **Supplemental Table 3. Primer sequences and PCR conditions for bisulfite pyrosequencing analysis** | | | | |
| --- | --- | --- | --- | --- |
| Gene Name | Forward Primer (5’🡪3’) | Reverse Primer (5’🡪3’) | Sequencing Primer (5’🡪3’) | PCR Cycling Conditions* |
| *TP53AIP1* | TGAGTAAGAAAAGGTGAGAAGAT | BTN-TTACCCTTTTCACAACCAATACT | TAATTTTTGTTGTATATATG | 94-59-72 x 55 |
| *SPATA21* | BTN-TTACCCTTTTCACAACCAATACT | CCTACCCTCACCTTCCTCTTAC | TCCTCTTACTTCAAAATCAATTA | 94-59-72 x 55 |
| *SOGA1* | TATTTAGGGTTGTGGTGTTGGTAG | BTN-CAAACAAACCTATCACCATTAAAA | GTTGTGGTGTTGGTAGTTA | 94-65/62-72 x 5 at each annealing temp, then 94-59-72 x 55 |
| *ADAM15* | BTN-AGAGTGAGGGGAGAGTTTGGTA | CCCCAACTCCTCCTATAAAAATTC | CAACAACAACTTATTCTAAT | 94-59-72 x 55 |
| *All reactions were performed with a heated lid at 99 degrees. Reactions began at 95 degrees for 15 minutes. Following 30 seconds at each cycling temperature, samples were heated at 72 degrees for 10 minutes and then cooled at 4 degrees. | | | | |
